# Platelet-rich plasma (PRP) as therapy for cartilage, tendon and muscle damage – German working group position statement

**DOI:** 10.1186/s40634-020-00282-2

**Published:** 2020-09-03

**Authors:** T. Tischer, G. Bode, M. Buhs, B. Marquass, S. Nehrer, S. Vogt, W. Zinser, P. Angele, G. Spahn, G. H. Welsch, P. Niemeyer, H. Madry

**Affiliations:** 1 Department of Orthopaedic Surgery, University medicine Rostock, Doberanerstr. 142, 18057 Rostock, Germany; 2 Klinik für Orthopädie und Unfallchirurgie, Universitätsklinikum Freiburg, Freiburg, Germany; 3 Norddeutsches Knorpelcentrum, COVZ Quickborn, Quickborn, Germany; 4 Klinik für Orthopädie, Unfallchirurgie und plastische Chirurgie, Universität Leipzig, Leipzig, Germany; 5 Donau University Krems, Krems, Austria; 6 Klinik für Sportorthopädie und arthroskopische Chirurgie, Hessing Stiftung, Augsburg, Germany; 7 Klinik für Orthopädie und Unfallchirurgie, St. Vinzenz-Hospital, Dinslaken, Germany; 8 Department of Trauma Surgery, University Medical Center Regensburg, Regensburg, Germany; 9 Center of Trauma and Orthopaedic Surgery Eisenach and Jena University Hospital, Jena, Germany; 10 UKE Athleticum, University Hospital Hamburg-Eppendorf, Hamburg, Germany; 11 OCM Gemeinschaftspraxis, Munich, Germany; 12 Center of Experimental Orthopaedics, Saarland University, Homburg, Germany

**Keywords:** Platelet-rich-plasma (PRP), Cartilage, Osteoarthritis, Tendon pathologies, Muscle injuries, Consensus statement

## Abstract

**Purpose:**

Platelet rich plasma (PRP) is widely used in orthopaedics, but is still heavily debated. Therefore, a survey among the German “Working Group for Clinical Tissue Regeneration” of the German Society of Orthopaedics and Traumatology was conducted to achieve a consensus about the current therapeutical potential of PRP.

**Methods:**

A first survey (*n* = 65 experts, all orthopaedic/trauma surgeons) was conducted (*n* = 13 questions). Following, a second round (*n* = 40 experts) was conducted with 31 questions to achieve consensus in 5 categories: three most common indications, PRP application, future research areas.

**Results:**

Therapeutic PRP application was regarded as useful (89%), possibly even more important in the future (90%). Most common indications were tendon pathologies (77%), osteoarthritis (OA) (68%), muscle injuries (57%) and cartilage damage (51%). Consensus was reached in 16/31 statements. The application of PRP for early knee OA (Kellgren-Lawrence grade II) was regarded as potentially useful, as well as for acute and chronic tendinopathies. For chronic lesions (cartilage, tendons), multiple injections (2–4) were seen preferable to singular injections. However, no sufficient data exists on the time interval between the injections. Standardization of PRP preparation, application, frequency, as well as determining the range of indication is strongly recommended.

**Conclusions:**

There is a need of further standardization of the PRP preparation methods, indication and application protocols for knee OA and other indications, which must be further evaluated in basic science studies and randomized controlled clinical trials.

**Level of evidence:**

Consensus of expert opinion, Level V.

## Background

Platelet rich plasma (PRP) is widely used in regenerative medicine and especially in orthopaedic sport medicine [14, 34, 45]. Basic science studies show many positive effects of PRP in vitro and in vivo on many cells of the musculoskeletal system, e.g. chondrocytes, tenocytes or muscle cells [5, 6, 21, 33, 41]. However, the quality of the available literature remains limited, both of basic science and clinical studies [15, 21, 34]. As a result, in clinical studies the effects are not as clear as in basic science studies [12, 26, 34].

Possible reasons for this are numerous. To start with, multiple preparation methods (currently over 25 different commercial systems available [13]) exist to obtain platelet-derived growth factors, but the final PRP products are limited by their inhomogeneous composition and their elaborative production protocols [4, 37, 45, 50, 51, 55, 59]. For example, different PRP preparation methods have shown different effects on articular chondrocytes [40]. Moreover, as the reporting of basic parameters like blood constituents (erythrocytes, leukocytes and thrombocytes) is still not performed in every study, there is an urgent need for a standardized reporting of these factors [13, 21]. Also there are large interindividual differences of the final PRP product [51]. To complicate matters even further, the dosing, timing and number of PRP applications are not standardized and not yet investigated sufficiently in basic science studies [61]. In this respect, the need for a standardized preparation of platelet-derived growth factors is obvious and would allow standardized basic science testing of the effect of different parameters like PRP preparation, amount of PRP injections and timing of injections [27, 36]. Further, the use of classifications to better describe the used PRP product should be mandatory. Several authors proposed different classification systems, among which are Mishra (platelet count, presence white blood cell, activation), Dohan Ehrenfest (platelet count, leukocyte count, presence of fibrin), Delong (**P**latelet count, **A**ctivation, **W**hite blood cells count; PAW classification) and Mautner (**P**latelet count, **L**eukocyte presence, **R**ed blood cell presence, and use of **A**ctivation; PLRA classification) [4]. Magalon et al. proposed the DEPA classification describing the **D**ose of injected platelets, **E**fficiency of production, **P**urity of the PRP and its **A**ctivation [49]. Harrision et al. published another comprehensive classification system including activation method if used, the total volume used, the frequency of dosing and subcategories of activation, platelet concentration and preparation technique and includes the overall average counts and range (low–high) of platelets, red cells and differential leukocyte counts (neutrophils, lymphocytes and monocytes) [28]. The most recent classification comes from Kon et al. based on an expert consensus and describing the most important factors to be reported as the platelet composition (concentration of platelets and concentration ratio), purity (presence of erythrocytes/leukocytes) and activation (endogenous/exogenous, addition of calcium) [38].

Many indications for PRP use are widely debated, e.g. the treatment of tendinopathies is complicated by the fact that clinical studies both describe positive and negative results for various locations [15, 22, 25, 31, 46, 48]. As a result, often no conclusive evidence can be drawn from the literature. This also makes it difficult for PRP therapy to be included in various guidelines. Since many open questions using PRP remain, the rationale of this paper was to display the current opinion of experts among the German “Working Group for Clinical Tissue Regeneration” of the German Society of Orthopaedics and Traumatology (DGOU) about the use of PRP and future research areas.

## Methods

**Fig. 1:**
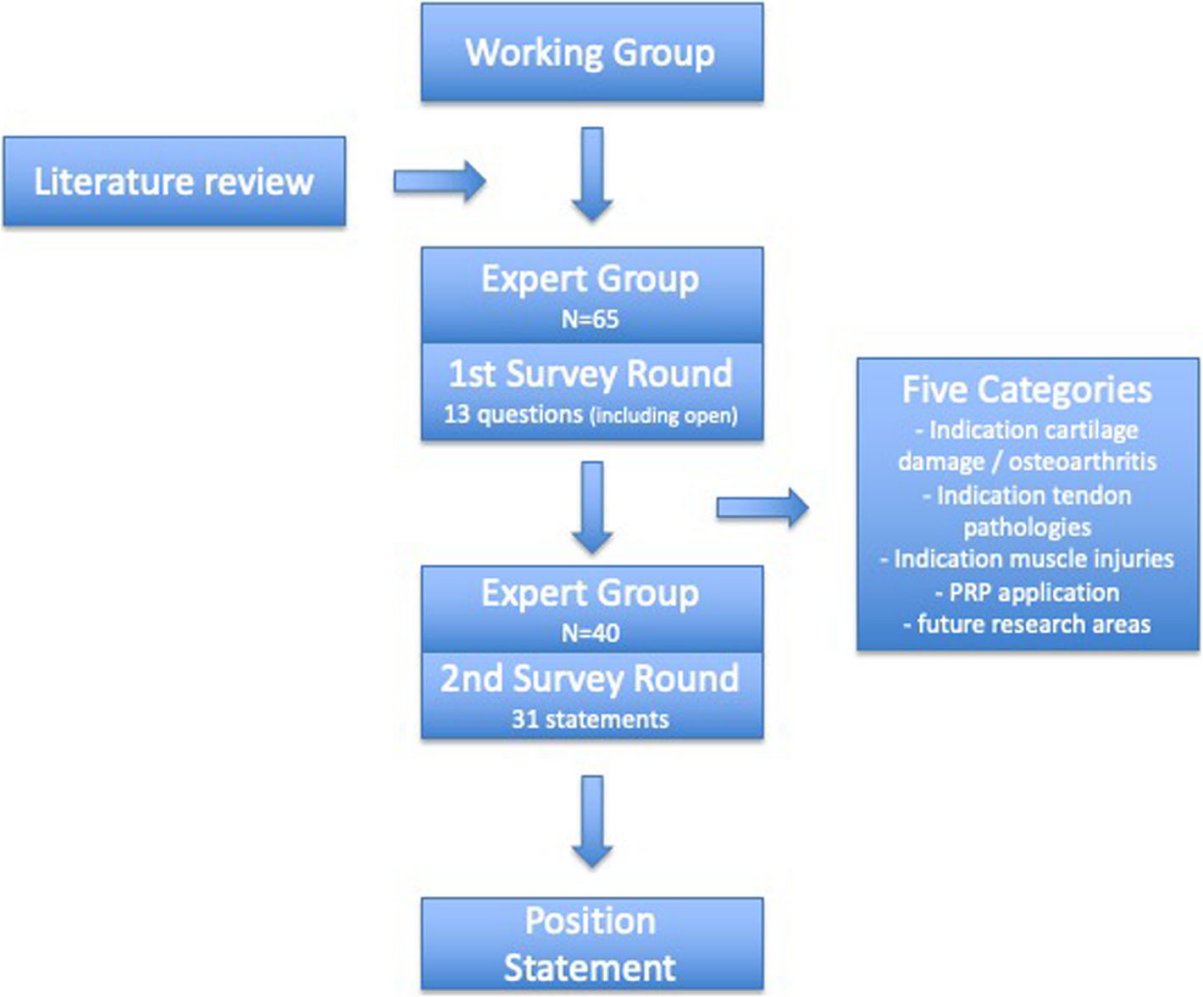
Flow of consensus process

The German “Working Group for Clinical Tissue Regeneration” consists of 95 members, each specialized in orthopaedic surgery and tissue regeneration (all MDs or PhDs, no physiotherapists or sports scientists). A working group of 5 individuals (blinded for review) was made responsible for facilitating the survey. Potential information items for inclusion within first-round survey were prepared by the working group after review of the available literature. A first survey was conducted in April 2018 with 13 questions regarding general aspects of PRP application with closed and open questions and experts were encouraged to propose further items or modifications. Resulting from these answers a second round was developed and conducted in November 2018 with 31 closed questions in five different categories: indication for cartilage damage and osteoarthritis (OA), indication for tendon pathologies, indication for muscle injuries, PRP application and future research areas. Figure 1 outlines the process used to develop the expert consensus.

Agreement was generated through online surveys (Survey Monkey, USA) to allow respondents to rate whether items should be included within minimum reporting requirements with five possible responses on a Likert [47] scale: “strongly agree”; “agree”; “neither agree nor disagree”; “disagree” or “strongly disagree”. The survey was piloted by three experts for face validity, understanding and acceptability, resulting in minor modifications. A total of sixty-five experts took part in the first round and forty experts took part in the second round. For consensus in round two, defined a priori, items were included in the final consensus document if over 75% of respondents agreed, and fewer than 20% disagreed. Agreement in 75% of participants is the most frequently specified determination of a consensus and was used in our study [19].

## Results

In the first round, 89% answered that PRP application is useful and 90% think that PRP will be even more important in the future. Most members are familiar with both basic science and clinical research, however only 58% use PRP in their daily practice. The most common reasons for not using PRP were no suitable setting, e.g. university hospital (41%), to expensive (19%), to time consuming (19%) or not enough scientific evidence (33%). The most common indications for PRP use were seen in tendon pathologies (77%), OA (68%), muscle injuries (57%) and cartilage damage (51%), which were the basis for the second survey round. Indications for intraoperative use of PRP were seen together with cartilage repair in 18% and tendon repair in 32%. Other indications were seen in 14%. Only 9% stated that there is no clinical use for PRP. PRP injections were sometimes combined with hyaluronic acid (11%). Besides PRP, the experts performed injections also with local anaesthetics (65%), cortisone (72%), hyaluronic acid (84%) and Traumeel/Zeel (28%). Furthermore, the experts overwhelmingly stated that more clinical studies about PRP application are required (76%) and also better standardization must be achieved (preparation 70%, indications 56%, timing of application 53%, number of injections 53%). Please see appendix for detailed information about the first round.

**Fig. 2:**
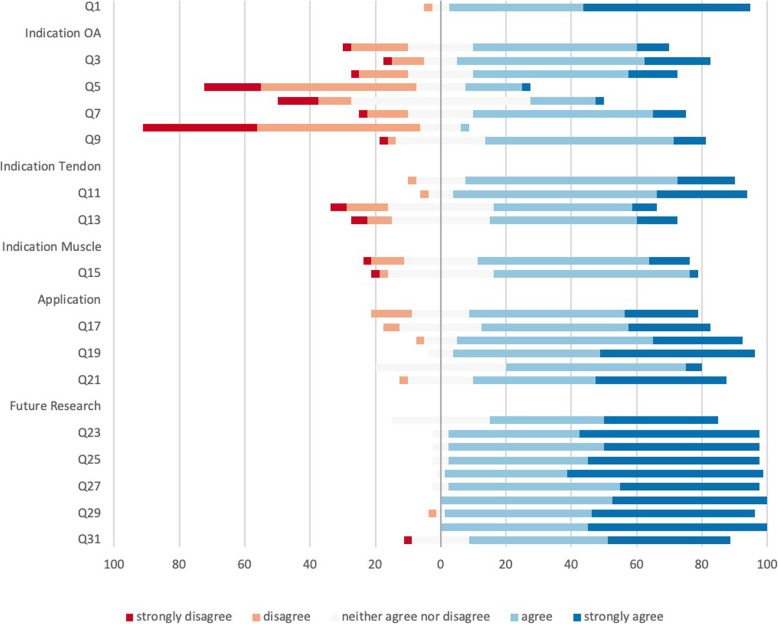
Stacked leaning bar chart representing breakdown in agreement levels in the second survey round (31 questions (Q1 – Q31)), showing the areas with disagreement nicely. Bars to the left of the Y axis indicate disagreement with bars to the right indicating agreement. Most disagreement is seen in the field of indications

Resulting from these answers the second round was more specialized in the topics of most interest. In 16/31 statements consensus was reached. Figure 2 also shows the areas where less consensus was reached, especially in the field of indication. It was commonly agreed (92%), that great differences exist in the various areas of indication for PRP applications (e.g. OA, tendinopathies, muscle injuries, ...).

### Indication for cartilage damage and OA

**Fig. 3:**
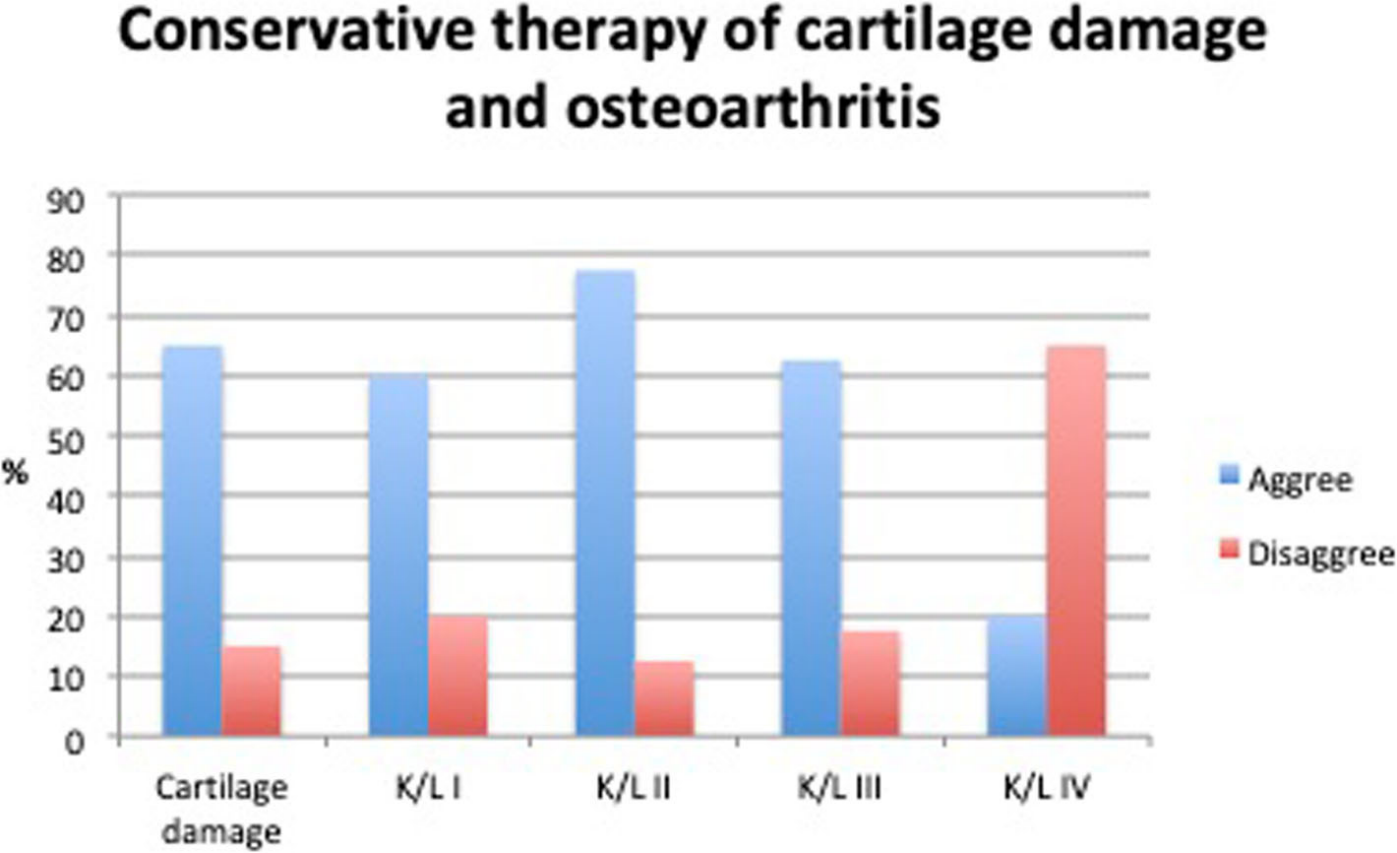
Recommended use of PRP for conservative therapy of cartilage damage and osteoarthritis

There was a common agreement (77.5%) that PRP may be used in early OA of the knee [Kellgren-Lawrence (KL) grade II] (Fig. 3). For less severe cartilage damage (KL grade I) and more severe stages (KL grade III and IV) no consensus was achieved. Also, no consensus could be achieved for intraoperative or postoperative use of PRP after cartilage regenerative surgery, although this was regarded as a promising field by 67.5% of experts (Table 1).

**Table 1:** Indications for use of PRP in cartilage damage and osteoarthritis

	% Agreement	% Dis-agreement
**Indication cartilage damage / osteoarthritis**		
The application for questionable OA (Kellgren-Lawrence grade I) can be useful	60,0	20,0
The application with minimal OA (Kellgren-Lawrence grade II) can be useful	77,5	12,5
The application for moderate knee OA (Kellgren-Lawrence grade III) can be useful	62,5	17,5
The application for severe knee OA (Kellgren-Lawrence grade IV) can be useful	20,0	65,0
The use of PRP preparations is preferable to hyaluronic acid preparations in the indication spectrum of incipient OA	22,5	22,5
The use of PRP for conservative therapy of cartilage damage (ICRS I/II) can be useful	65,0	15,0
The intraoperative use of PRP in cartilage regenerative surgery has been sufficiently proven to date	2,5	85,0
The postoperative use of PRP can be useful for promoting biological cartilage regeneration	67,5	5,0

### Indication for tendon pathologies

In the survey, the experts represent the opinion in large majority (82.5 and 80%) that the use of PRP in acute and chronic tendinopathies can be useful. In cases of rotator cuff repair, 50% of experts are of the opinion that an intraoperative application of PRP is potentially useful, however 17.5% of them have a contrary opinion. A similar number of experts (57.5%) believe in a positive effect of PRP in the postoperative treatment after tendon repair (Table 2).

**Table 2:** Indication for use of PRP in tendon pathologies

	% Agreement	% Dis-agreement
**Indication tendon pathologies**		
The application for acute tendinopathy can be useful	82,5	2,5
The application for chronic tendinopathy can be useful	80,0	2,5
Intraoperative use to improve tendon healing (e.G. *rotator* cuff, Achilles tendon, ...) can be useful	50,0	17,5
The application for postoperative biological improvement of healing after tendon sutures can be useful	57,5	12,5

### Indication for muscle injuries

No consensus (e.g. an agreement over 75%) for the use of PRP for either acute or chronic muscle injuries was found, although the majority of experts supported the use of PRP (Table 3).

**Table 3:** Indication for use of PRP for muscle injuries

	% Agreement	% Dis-agreement
**Indication muscle injuries**		
The application for acute muscle injuries can be useful	70,0	12,5
The application for chronic muscle injuries can be useful	62,5	5,0

### Practical aspects of PRP application

Agreement could be achieved for the following three statements: (1) that chronic lesion require more than one PRP injection, (2) that there is no sufficient information regarding the best time interval in between injections (no consensus was found for weekly intervals) and (3) that the variability of the different PRP preparations may play an important role for its biological effects (Table 4).

**Table 4:** Practical aspects of PRP application

	% Agreement	% Dis-agreement
**PRP application**		
When using PRP, the injection is isolated (subcutaneous wheals with LA excluded)	70,0	12,5
The frequency of use varies for the different indications (cartilage, tendon, muscle)	70,0	5,0
For chronic lesions, multiple injections (2–4) at intervals are preferable to singular injections	87,5	2,5
There is no sufficient data on the time interval between the individual injections	92,5	0,0
Weekly intervals between injections are preferable	60,0	0,0
The variability of the individual PRP composition plays an important role for the effect of PRP	77,5	2,5
There are differences in the effectiveness of PRP products depending on the manufacturing process	70,0	0,0

### Future research areas

Consensus was achieved on all statement about future research areas (Table 5). The PRP production must be better standardized (95% agreement) as well as its clinical application (e.g. injection frequency, application time, clinical indications). Even in fields where there is supposedly good clinical data like in the treatment of OA, the expert members felt there is still a great need for more basic science and clinical studies. The same holds true for the other indications.

**Table 5:** Main future research areas

	% Agreement	% Dis-agreement
**Future research areas**		
The production of PRP must be better standardised	95,0	0,0
The use (injection frequency) of PRP needs to be better standardised	95,0	0,0
The application (application time) of PRP must be better standardised	95,0	0,0
The range of indications for PRP must be better standardised	97,5	0,0
For use in the indication field of the beginning arthrosis, further basic studies are necessary	95,0	0,0
For the application in the indication field of incipient arthrosis further clinical studies are necessary	100,0	0,0
Overall, further basic studies are also required for the other indication areas	95,0	0,0
Overall, further clinical studies are also required for the other indication areas	100,0	0,0
In the future, PRP will play an important role in biological therapeutic procedures	80,0	2,5

## Discussion

The results of our study show, that the topic of PRP application in orthopaedics is still widely debated, even among a national group of specialists. In only 16 of 31 statements a common consensus was reached. Greatest consensus existed in the field of future research areas, suggesting that there is a strong need to generate extended evidence by performing many different future studies. In this regard, a critical appraisal of available evidence by specialist working groups is one way to improve medical knowledge [2, 30].

### Indication for OA and cartilage damage

In accordance with current literature, PRP may be indicated for early and moderate forms of OA [7, 24, 29, 43, 53]. Newer evidence shows that the intra-articular administration of PRP might also improve the symptoms of the patient, regardless of the degree of cartilage damage, however good subgroup analysis according to the Kellgren and Lawrence classification are often lacking [7, 11]. In this context, experts currently do not recommend to use PRP for KL grade 4 due to the insufficiently available data. PRP also has the potential to improve knee function, possibly by reducing the inflammatory response and slowing the degenerative remodeling process of the articular cartilage. Better results with PRP are generally achieved in patients that are male, young, with a lower degree of cartilage damage and with low body mass index (BMI).

The composition of PRP appears to be a crucial parameter when interpreting the published clinical data. Since a cytotoxic effect of leukocyte rich plasma on synoviocytes has been shown in vitro, LP-PRP is mostly recommended for intraarticular application [9]. In a recent basic science study comparing leukocyte poor (LP) and leukocyte rich (LR) PRP on OA development in a post-meniscectomy model in mice, LP-PRP showed superior results in preserving cartilage volume than LR-PRP [32]. A recent meta-analysis of randomized controlled trials found PRP superior in outcome compared to hyaluronic acid (HA) with a subgroup analysis showing better results of LP-PRP compared to LR-PRP, however without directly comparing LR- and LP-PRP, making further studies necessary [7]. In fact, the largest study comparing LR-PRP and HA shows no detrimental effect of LR-PRP [17]. Also, a clinical study directly comparing LR- and LP-PRP shown no clinical difference in outcome after 12 months [23]. LR-PRP contains more proinflammatory molecules and a higher concentration of growth factors but also a higher concentration of anti-inflammatory cytokines like interleukin 1 receptor antagonist (IL1-Ra) [64]. Newer studies describe the process of “inflammatory regeneration” with the secretion of both pro- and anti-inflammatory cytokines by the leukocytes, showing positive effects on tissue regeneration [42]. Additional clinical studies with prospectively randomized design are required to identify parameters of optimal production or composition of the PRP preparations and an ideal application protocol in OA [18, 29, 43, 44, 53, 61].

In accordance, it was suggested that both HA and PRP may be a superior treatment for patients with mild OA and a low BMI [16, 17]. Recent systematic reviews show a greater therapeutic efficacy of PRP compared to HA [7]. However, open points raised consistently included the need of standardized PRP preparation, the rate of application and the need of further randomized clinical trials with a high level of methodological quality. Therefore, official recommendations and guidelines are currently often inconclusive in terms for or against the use in knee OA. In conclusion, on the basis of current evidence with the restriction of a high methodological variability by different preparation protocols, PRP might lead to a pain improvement in mild to moderate OA. This expert group does not recommend PRP in cases of severe OA. Newer studies show that PRP also contributes to a placebo effect, which has been shown especially in the treatment of OA [24] or lateral epicondylitis [34]. PRP injection may be just one part of an overall therapeutic treatment strategy, addressing biological issues of OA. In addition to other important factors such as weight reduction, malalignment correction, muscle training, knee braces and others it might be helpful in achieving pain reduction and a better outcome for the patient.

The role of PRP in regenerative cartilage surgery is another widely debated field [21, 35]. Although basic science studies show positive effects on chondrocytes [21, 27, 33, 40], clinical evidence for using PRP intraoperatively, at the time of cartilage regenerative surgery or in the rehabilitation phase is still insufficient, mirroring the results of our survey. Furthermore, the optimal timing of PRP treatment after the surgical procedure remains uncertain. But most experts agree that PRP can potentially be useful for promoting biological cartilage regeneration. Taken together, the results of the present critical judgement show that a possible role of PRP in regenerative cartilage surgery must be further evaluated.

### Indication for tendon pathologies

The use of PRP for the treatment of tendinopathy is a topic greatly debated in the literature. A review about basic science studies showed positive in vitro (e.g. increased tenocyte proliferation, positive anabolic effects like increased collagen production) and in vivo (increased tendon healing) effects of PRP [63]. Clinically, there are currently many studies showing both positive effects but also no effects for PRP treatment in various tendon disorders, both acute and chronic [12, 14, 15, 22, 54]. As one example, a recent systematic review has highlighted the controversial results of PRP applications for different tendon pathologies with mainly positive effects on lateral elbow tendinopathy and patellar tendons but not Achilles tendons or rotator cuff pathology, where the vast majority of surgical RCTs documented a lack of beneficial effects, whereas there is still inconclusive evidence concerning its conservative application in rotator cuff disorders [22]. For lateral epicondylitis current meta-analyses show short term positive effects of corticosteroids but superior long-term effect of PRP [31, 34, 46]. Following current evidence, patellar and lateral elbow tendinopathy showed improvement following PRP treatment while the Achilles tendon and rotator cuff do seem not to benefit from PRP application. A recent consensus of the ESSKA basic science committee therefore concluded about the use of PRP for tendinopathy, that there is currently no consensus [2]. Although debated in the literature, as newer studies and systematic reviews show, there are both from a basic science as well as clinical perspective, positive effects of PRP in the treatment of tendon disorders. Especially when accounting for the possible side effects of corticosteroids in the use of tendiopathy. The results of this survey showed, that it is the current opinion in Germany, that PRP can be useful for treatment of both acute and chronic tendinopathies.

### Indication for muscle injuries

Even more debated is the use of PRP for muscle injuries, which are one of the most common lesions in professional sport therefore responsible for about 30% of the days off the pitch [20]. Offering a possibility to improve biological healing and accelerate the return to sport rates, PRP has been of growing interest in the past few years. Even though 57% of given answers in the first round named muscular injuries among the most common indication for PRP use, solid scientific background is still lacking. Several in vitro studies observed potential benefits of PRP use in muscular injuries. Acceleration of satellite cells activity, increased diameter of regenerated fibrils, stimulation of myogenesis and increased activity of MyoD and Myogenin are well examined [39]. Further on Mazzocca et al. observed increased concentration of growth factors such as HGF, FGF and EGF for PRP-LP [52]. These findings were underlined by Tsai et al. proving increased skeletal muscle cell viability and cell proliferation by shifting cells from the G1 phase to S1 phase and G2&M phases after PRP application, besides demonstrating increased protein expressions of cyclin A2, cyclin B1, cdk2 and PCNA [60]. A recent systematic review summed up the current scientific background as follows: (1) in the majority of studies, PRP treatment increased myocyte proliferation, growth factor expression (e.g., PDGF-A/B and VEGF), leukocyte recruitment, and angiogenesis in muscle models when compared to control groups; (2) PRP preparation techniques remain inconsistent across studies in the basic science literature; and (3) evidence from in vitro and in vivo basic science studies suggest that PRP has the potential to serve as an efficacious treatment modality that may expedite the healing process for muscular pathologies, based on observed effects at the cellular and tissue levels in treatment groups relative to control groups [41].

While retrospective studies described complete healing and no significant advantage considering time off the pitch [57], Bubnov et al. observed less pain and significantly faster return to play in a cohort study of 30 athletes [10]. Hamid et al. described significantly faster return to play in a randomized controlled trial (RCT) comparing PRP infiltration versus a conservative treatment protocol the only double/blinded multicenter RCT including hamstring injuries in athletes (*n* = 80) did not observe any significant results for PRP in comparison to placebo infiltration [1]. The described above promising biological rationale, the positive preclinical findings, and the successful early clinical experience of PRP injections are not confirmed by the recent high-level RCTs [26]. A current consensus among members of the GOTS evaluated conservative therapies for muscle injuries and concluded that there is presently no clear evidence that intramuscular injections are of use in the treatment of muscle injuries [30]. This is in line with our results, where no agreement could be achieved on the use of PRP for muscle injuries. Further studies investigating dosage, time and frequency of PRP in muscular injuries are highly required. Comparably to cartilage damage, also in muscle injuries, the treatment algorithm and especially the use of PRP might be correlated to the grade and chronicity of the injury, differentiating in between the involved amount of the injured muscle diameter and possible tendon lesions or avulsion injuries.

### PRP application

The field of PRP application is one of the most commonly discussed field and lacking standardization is one of the major problems of current clinical trials. Most experts use PRP without any additions, however there are some studies suggesting that the additional use of hyaluronic acid might be advantageous over single use of PRP for OA [56, 58, 62]. Consensus was reached that more than one injection should be performed for chronic lesions, a recommendation supported in the field of OA, where multiple injections show better results than singular injections [61]. Basic science studies are exploring the dose - effect relationship of PRP, but these results have still to be transferred to clinical studies [27]. The optimal concentration of PRP has not yet been identified, and studies describing that higher concentrations might have negative effects [8]. Similarly, the effects of leukocytes is dependent on the indication, with some indications requiring leukocyte poor PRP. The variability of the individual PRP composition plays an important role for the effect of PRP.

### Future research areas

Agreement was that more research about PRP must be conducted in the future, in line with recent publications [13]. One of the major problems is that PRP preparations must be better standardized (95% agreement). One possible aspect toward this goal could be the pooling of platelets in order to achieve more volume, which is more standardized [3, 27, 36]. Also, various parameters for clinical application are unknown, like how many injections should be used, the time in between injections as well as the dosing of PRP. Only then it is possible to perform high-level studies and evaluate which indications are best for the use of PRP, making both basic science as well as clinical studies, preferably randomized-controlled studies, necessary. Although consensus was reached that PRP may play an important role in the future, right now it appears that more experimental and clinical studies are needed.

#### Limitation

A possible limitation of this survey trying to address the widely debated topic of PRP application is its national character. The national differences in availability and reimbursement of PRP might influence the results, as well as regulatory aspects. Further, the consensus was not multidisciplinary, incorporating only the views of orthopaedic surgeons. However, this may be also viewed as a strength, since this is the only group that actively carries out and supervises PRP injection therapies. Moreover, the performed survey has not the same methodological quality as a rigorously performed Delphi process. A strength is the consensus formed by a group of specialized orthopaedic surgeons with a large body of expertise in their respective fields, both from a basic scientific and clinical point of view.

## Recommendations

Based on the agreement in at least 75% of the participating experts a consensus was defined for the following points:

- OA and cartilage damage: The application with mild knee OA (KL grade II) can be useful
- Tendon pathologies: The application for acute and chronic tendinopathies can be useful
- Practical recommendations: For chronic lesions (cartilage, tendons), multiple injections (2–4) at intervals are preferable to singular injections. There is however no sufficient data on the time interval between the individual injections
- Future research: Standardization of PRP production, preparation, application, frequency, as well as range of indication is strongly recommended. Further basic and clinical studies are necessary.

## Conclusion

The common agreement was that differences exist in the various areas of indication for PRP applications, and that there is still a great uncertainty in the standardization of the PRP procedure itself, especially for the different indications. PRP application in cases of early OA of the knee (KL grade II) may be useful, as well as for acute and chronic tendinopathies. For chronic (cartilage and tendon) lesions, multiple injections (2–4) at intervals are preferable to singular injections, but there is not sufficient data on the time interval between the individual injections. One major problem is the variability of the individual PRP composition, which plays an important role for the effect of PRP. Therefore, the production of PRP must be better standardized, as well as clinical parameters like the number of injections, the time in between injection and exact indications. Even for OA, currently representing the best investigated field for PRP application, more basic science and clinical studies are necessary, as well as for the presented other indications.

## Supplementary information

**Additional file 1.**

## Data Availability

Not applicable.
