# Supplementary material for: Platelet-rich plasma (PRP) as therapy for cartilage, tendon and muscle damage – German working group position statement
Source: J Exp Orthop. 2020 Sep 3;7:64. doi: 10.1186/s40634-020-00282-2 (PMC7471237; doi:10.1186/s40634-020-00282-2)
Supplement: Supplementary file 1 — Additional file 1. [file 40634_2020_282_MOESM1_ESM.docx]

Supplement

Questions of survey round 1:

1. Do you currently consider the use of PRP for certain indications to be useful ?

Yes 89.2 %, No 10.8 %

2. Do you see a future potential for increased use of PRP ?

Yes 90.6%, No 9.4%

3. Are you familiar with the current basic research on PRP?

Very well familiar 13.9%, Well familiar 46,2%, Somewhat familiar 38,5%, Not familiar at all 1.5%

4. Are you familiar with the current clinical study situation on PRP ?

Very well familiar 15.4%, Well familiar 49,2%, Somewhat familiar 33,9%, Not familiar at all 1.5%

5. Do you use PRP in clinical practice ?

Yes 58.5%, No 41.5%

6. If not, why not? (multiple choice possible)

no sufficient scientific evidence 33.3%

too expensive 18.5%

no corresponding setting (e.g. university) 40.7%

too time-consuming 18.5%

haven't had time to deal with it yet 3.7%

different reasons 25.9%

7. For which indications do you see a reasonable use for PRP ?(multiple answers possible)

In addition to cartilage regenerative measures intraoperative 18.5%

In addition to cartilage regenerative measures postoperative 50.8%

Conservative therapy in osteoarthritis 67.7%

Tendon attachment irritation (e.g. epicondylitis, patellar tip syndrome etc.) 76.9%

Muscle injuries 56.9%

Promotion of postoperative healing (except cartilage, e.g. tendon sutures) intraoperative 32.3%

Promotion of postoperative healing (except cartilage, e.g. tendon sutures) postoperative 50.8%

Other indications 13.8%

No practical use of PRP 9.2%

8. If other indications, which ?

Fracture healing, meniscus degeneration, wound healing problems, ACL healing

9. What are the most important measures to establish PRP in clinical practice ? (multiple answers possible)

Better standardization of PRP production (platelet content, leucocyte content, concentration of active factors in the final product) 70.3%

Better standardization of the application (injection frequency) 53.1%

Better standardization of application (timing of injection for postoperative use) 53.1%

Better standardization of indications 56.3%

More clinical studies 76.6%

More basic studies in vitro 35.9%

More basic studies in animal models 28.1%

Sufficiently good studies available, no further measures necessary 1.6%

Secured reimbursement of costs by health insurance companies 39.1%

Other measures 1.6%

10. What other measures that are important for the establishment of PRP can you think of ?

Simple handling, clear indications

11. What would be of particular importance to you regarding the question "Status of the application of PRP"?

More evidence, more prospective randomized studies, dosing and timing of injections

12. Do you use PRP isolated or in combination with other active substances/measures (multiple answers possible) ?

Isolated 83.0%, with Hyaluron 10.6%, with Local anesthetic 4.2%, with Glucocorticoid 2.1%, with other substance 8.5%

13. Do you use any other drugs besides PRP for injection therapy (multiple answers possible) ?

Local anaesthetic 64.9%, cortisone 71.9%, Hyaluronic acid preparations 84.2%, Traumeel/Zeel 28.1%, Actovegin 1.7%, Other medications 3.5%
